# Relapsing subarachnoid hemorrhage as a clinical manifestation in microscopic polyangiitis: a case report and literature review

**DOI:** 10.1007/s10067-022-06163-6

**Published:** 2022-06-11

**Authors:** Jingjing Xie, Ertao Jia, Suli Wang, Ye Yu, Zhiling Li, Jianyong Zhang, Jia Li

**Affiliations:** 1 The Fourth Clinical Medical College of Guangzhou University of Chinese Medicine, No.1, Fuhua Road, Futian District, Shenzhen, Guangdong, 518033 China; 2 The Department of Rheumatology, Shenzhen Traditional Chinese Medicine Hospital, No.1, Fuhua Road, Futian District, Shenzhen, Guangdong, 518033 China; 3 Department of Rheumatology, Ren Ji Hospital, Shanghai Jiao Tong University School of Medicine, Shanghai, 200001 China

**Keywords:** Anti-neutrophil cytoplasmic antibody (ANCA)-associated vasculitis, Microscopic polyangiitis, Subarachnoid hemorrhage

## Abstract

Microscopic polyangiitis (MPA) is a systemic small-vessel vasculitis associated with anti-neutrophil cytoplasmic antibody (ANCA) and predominantly causes kidney and pulmonary injuries. Subarachnoid hemorrhage, a life-threatening manifestation of the central nervous system (CNS), rarely occurs in patients with ANCA-associated vasculitis (AAV). We report the case of a young man with spontaneous SAH recurrence and active nephritis. The patient was treated with a glucocorticoid pulse and intravenous cyclophosphamide (CTX) in combination with decreasing cerebral perfusion pressure and analgesic therapy. All the patients’ symptoms except the proteinuria resolved. We reviewed the clinical characteristics of 34 previously reported cases of SAH with AAV, comprising six cases of MPA, eight cases of granulomatosis with polyangiitis (GPA), and 19 cases of eosinophilic granulomatosis with polyangiitis (EGPA), and one case of unclassified AAV. All the cases showed features of active vasculitis. Concomitant nephritis and peripheral neuropathy were found in the MPA and EGPA cases with SAH, respectively. Renal and pulmonary manifestations were predominant in the patients with GPA and SAH. Ten patients had aneurysmal abnormalities, and six patients had cardiac abnormalities. Thirty-one patients were treated with glucocorticoids, and 18 patients received concurrent immunosuppressants. Patients with SAH had a mortality rate of 38.2%. The presence of cerebrovascular events or cardiac involvement in patients with AAV and SAH is associated with increased mortality of 64.3%. Our study indicates that SAH should be cautioned as a disease occurring in patients with AAV. Early diagnosis with aggressive immunosuppressive therapy can help improve the prognosis of patients with SAH.

**Supplementary information:**

The online version contains supplementary material available at 10.1007/s10067-022-06163-6.

## Introduction

Anti-neutrophil cytoplasmic antibody (ANCA)-associated vasculitides (AAV) are a group of systemic necrotizing vasculitides that affect predominantly small vessels such as capillaries, venules, and arterioles. AAV includes granulomatosis with polyangiitis (GPA), microscopic polyangiitis (MPA), and eosinophilic granulomatosis with polyangiitis (EGPA). It is more common in those aged over 60 years males, especially in East Asiaz [1]. The clinical manifestations of AAV largely depend on the affected vasculature. The lungs, kidneys, and skin are typically affected organs [2]. CNS manifestations have rarely been reported [3] in patients with AAV. A few cases have demonstrated stroke, hypertrophic pachymeningitis, massive intracerebral hemorrhage (ICH), SAH, and spinal SAH [4, 5] in MPA. EGPA presents with four distinct neurological characteristics, including cerebral ischemic lesions, ICHs, cranial nerve palsies, and loss of visual acuity [6]. CNS involvement is characterized by pachymeningitis, cerebral ischemic lesions, hemorrhagic lesions, and hypophyseal lesions in patients with GPA [7]. Here, we report a patient who presented with SAH as the initial symptom of MPA and who experienced a relapse of SAH. We also reviewed the clinical characteristics of 34 previously reported cases of AAV with SAH.

### Case presentation

**Fig. 1:**
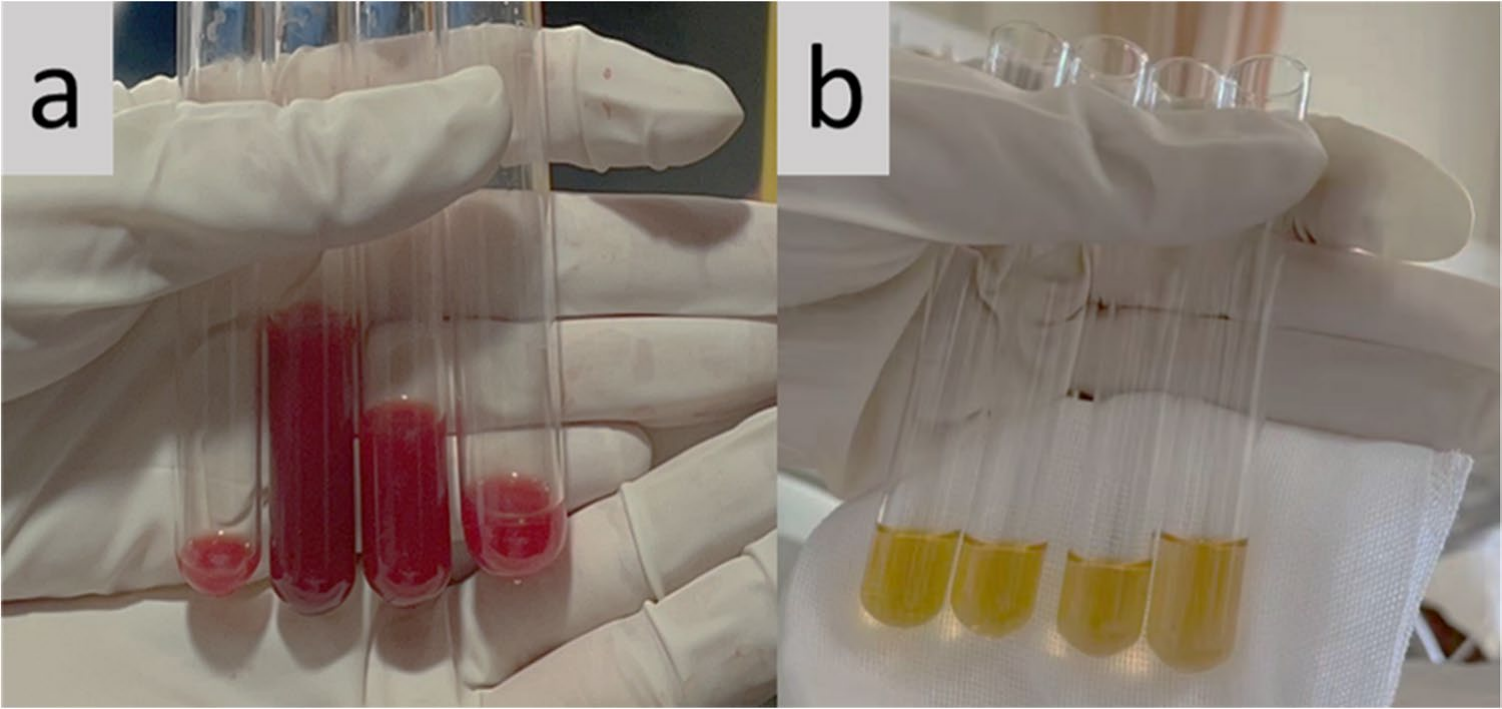
**a** Cerebrospinal fluid examination showed bloody fluid at the visit. **b** Xanthochromia in the cerebrospinal fluid was detected after treatment

A 31-year-old male with a 2-day history of acute headache and fever was admitted to our department. He complained of “tearing” back pain following a sudden sneeze, neck rigidity, and diffuse back pain, preventing him from lying down. The patient had no history of trauma or hypertension. Physical examination revealed blood pressure of 123/83 mmHg and a regular pulse rate of 82 beats/min. The patient had nuchal rigidity with positive Kernig’s and Brudzinski’s signs. Laboratory examinations revealed a white blood cell (WBC) count of 13.22 × 10^9^/L (normal range: 3.5–9.5 × 10^9^/L), a hemoglobin level of 151 g/L, and a platelet count of 293 × 10^12^/L. Urinalysis showed proteinuria (24-h urine protein 5.9 g), microscopic hematuria (urine sediment red blood cells 254.0/μL), and cast (pathological renal tubules of 1.0/μL). The creatinine level was 115.0 μmol/L (normal range: 57–97 μmol/L), eGFR level was 72.6 mL/min, with an increased erythrocyte sedimentation rate (ESR) of 34 mm/H (normal range: 0–20 mm/H) and a slightly increased C-reactive protein (CRP) of 12.7 mg/L (normal range: 0–10 mg/L). The tests revealed a positive p-ANCA and an elevated myeloperoxidase-ANCA (anti-MPO) level of 3.26 (normal range: normal < 1). Pulmonary computed tomography (CT) scan revealed multiple focal emphysema in bilateral lungs, bullae, and tiny ground-glass nodules in the lower lobe of the right lung. Brain CT and magnetic resonance imaging/magnetic resonance angiogram did not show bleeding, aneurysm, or malformation. A lumbar puncture revealed bloody cerebrospinal fluid (CSF) (Fig. 1a), with a pressure of 180 mmH_2_O, elevated protein of 2708.8 mg/L, a glucose level of 0.79 mmol/L, whereas blood glucose level of 4.1 mmol/L, chloride level of 121.0 mmol/L, red blood cell of 52,200 × 10^6^/L, and normal WBC count. Cerebrospinal fluid x-pert and metagenomic next-generation sequencing (mNGS) were negative, ruling out the presence of CNS infection. He was diagnosed with SAH.

**Fig. 2:**
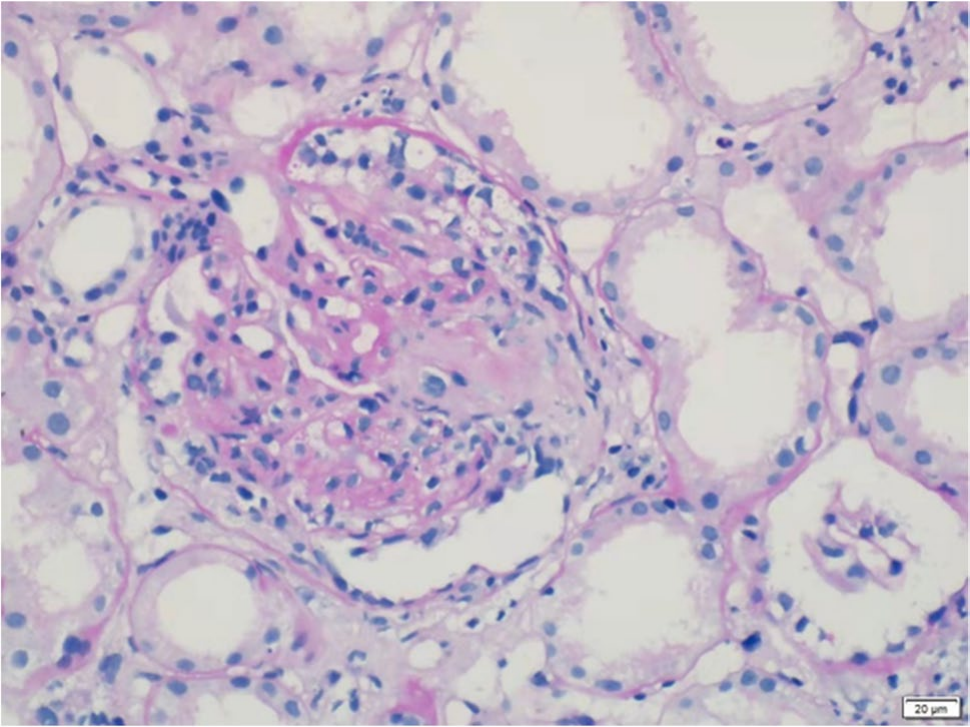
Renal biopsy showed global (4/23) or segmental (6/23) glomerulosclerosis, focal segmental necrotizing glomerulonephritis with endocapillary lesions, fibrocellular crescents (10/23), and accompanied by marked tubulointerstitial inflammation

Seven years earlier, the patient presented with a severe headache and vomiting. On examination, his blood pressure was 135/75 mmHg, and his pulse rate was 78 beats/min. Laboratory testing showed an increased CRP of 53.9 mg/dL and an ESR of 70 mm/h. Urinalysis showed microscopic haematuria (2 +) and proteinuria (1.78 g/24 h). Emergent cranial CT revealed SAH, and cerebral digital subtraction angiography was performed, which did not reveal any aneurysms or arteriovenous malformations. Based on his positive MPO-ANCA and renal biopsy findings with pauci-immune necrotizing glomerulonephritis and tubulointerstitial inflammation (Fig. 2), a diagnosis of MPA was made, in accordance with 2012 revised International Chapel Hill Consensus Conference Nomenclature of vasculitides [8]. The patients’ spontaneous intracranial SAH was attributed to MPA. He was administered prednisone (60 mg/day) combined with intravenous pulse CTX administration for 6 months and then switched to mycophenolate mofetil (MMF 1.0 g/day) for maintenance immunosuppression. The patient achieved complete remission in 12 months with normal urinalysis and serum creatinine level without neurologic sequelae.

In conjunction with the medical history of the patient, the recurring symptoms of fever and an increase in the urine protein, ESR, CRP, and MPO-ANCA were attributed to active vasculitis. The patient was given intravenous methylprednisolone (0.5 g) daily for 3 days and then tapered to oral prednisone at a dose of 1 mg/kg/day combined with nimodipine and analgesic therapy, followed by an intravenous injection of 0.8 g CTX. His symptoms were relieved within 2 weeks. The second lumbar puncture showed yellow cerebrospinal fluid (Fig. 1b), a pressure of 120 mmH2O, protein of 1056.3 mg/L, glucose level of 3.47 mmol/L, chloride level of 111.0 mmol/L, and normal WBC count. The patient was discharged from the hospital with no neurological symptoms. During the 6-month follow-up, the patient was treated with intravenous CTX every month with prednisone tapering to 10 mg/day. The patient was in good condition, and all symptoms except proteinuria had resolved.

### Literature review

The review is based on a literature search of PubMed, Web of Science, and Embase databases up to December 2021. The following MeSH terms or keywords were used: “microscopic polyangiitis,” “granulomatosis with polyangiitis,” “Churg-Strauss syndrome,” “eosinophilic granulomatosis with polyangiitis,” “anti-neutrophil cytoplasmic antibody-associated vasculitis,” and “subarachnoid hemorrhage” without language restrictions. Case reports and case series of patients with the diagnoses of AAV and SAH were eligible for inclusion. Publications were excluded if they did not meet the above criteria, if AAV overlapped other connective tissue diseases, or if they were review articles with no clinical case reports. Our literature review identified 143 citations, 73 were not relevant, and 37 were duplicate records. Ultimately, we included 33 reports with 34 cases.

## Discussion

The clinical presentation of AAV depends on the affected vessels, with mostly kidney and lung involvement. Mononeuritis multiplex [1] is the most frequent neurological manifestation of AAV. In contrast, CNS involvement is uncommon, with 5–15% in AAV [9] and 2–8% in MPA [10], including cerebrovascular events, such as hypophysitis, posterior reversible encephalopathy syndrome, isolated mass lesions, hypertrophic pachymeninges, and spinal cord lesions [11]. A retrospective study found that cerebral ischemic lesions were the main manifestations in Chinese patients with AAV [12]. The co-occurrence of AAV and SAH is uncommon and has not been fully elucidated. We describe a rare manifestation of MPA in a young man who presented with relapsing SAH. The patient had no history of hypertension, aneurysm, or arteriovenous malformations, without an increased risk of SAH. SAH was considered to be due to active vasculitis. He received a glucocorticoid pulse and intravenous CTX in combination with decreasing intracerebral hemorrhage (ICH) therapy, achieving remission at follow-up.

A noncontrast CT scan is a sensitive method to identify patients with subarachnoid hemorrhage. But CT imaging depends on patients presenting within 6 h of onset of acute headache and exhibits inadequate sensitivity to detect spontaneous SAH [13]. Lumbar puncture has been found to show evidence of hemorrhage in 3% of patients with a normal head CT [14]. Four of the 34 cases with negative CT were confirmed to have SAH using a lumbar puncture.

Interestingly, the patient showed lower glucose levels in CSF, which frequently accompany intracranial infection. However, an extensive evaluation, including mNGS of CSF, excluded the diagnoses of infection. Hypoglycorrhachia in CSF following SAH has seldom been reported and is associated with multiple reasons [15]. Alterations in the carrier transport system of glucose in and out of the CSF, caused by diffuse meningeal inflammation, increase anaerobic glycolysis. Vasospasm accounts for a decrease in CSF glucose levels.

We reviewed the literature and summarized the clinical characteristics and treatment of 34 cases with SAH (Table 1). Among the 34 cases, six were attributable to MPA, eight to GPA, 19 to EGPA, and one to unclassified AAV. Their ages ranged from 17–85 years, and 55.9% of them were women. The disease duration was up to 20 years. Three patients presented with SAH as the initial symptom of AAV. Three patients experienced a recurrence of SAH. Nephritis was the major non-CNS system disorder in MPA. EGPA was associated with more concomitant peripheral neuropathy. Renal and pulmonary manifestations were more common in patients with GPA and SAH. Ruptured saccular aneurysms are the main cause of nontraumatic SAHs [12]. As illustrated by the cases, only ten patients with aneurysmal SAH and two patients with intracranial artery dissection had similar incidences in different types of AAV. All the cases appeared to have evidence of active vasculitis, organ or life-threatening features, including active glomerulonephritis, progressive peripheral or cranial neuropathy, and gastrointestinal and cardiac disease due to vasculitis. Other manifestations included arthritis, myalgia, rhinosinusitis, skin vasculitis, pulmonary nodules, and asthma. They were accompanied by enhanced high-titer ANCA, elevated inflammatory factors, or increased eosinophilic granulocytes.

**Table 1:** Clinical characteristics and treatment of previously reported AAV patients with SAH

Author	Age	Sex	Disease duration	Dx	ANCA	CT/ MR	Aneurysm		CNS	SI	Biopsy tissue	Steroid	IS agents	Relapse	Outcome
Sae Aratani et al. 2017^[16]^	54	M	1 month	MPA	MPO	+	−		SAH Cerebral infarction	Renal Unstable angina	-	MP 0.5 g	-	-	Death
Xia Wang et al. 2015^[17]^	24	M	Present	MPA	P-ANCA MPO	Initial-relapse +	−		SAH	Renal	Renal	PNL 30 mg	MMF	SAH	Remission
Hidehito KIMURA et al. 2012^[18]^	44	F	3 years	MPA	MPO	+	+		SAH	Renal Pulmonary	Renal	MP pulse	CTX	Aneurysm	Remission
Baldwin L et al. 2001^[19]^	78	M	NR	MPA	p-ANCA	+	−		Spinal SAH Late-onset cerebellar ataxia	Renal	Autopsy	NR	NR	-	Death
Katsuhito Ihara et al. 2019^[20]^	85	F	Present	MPA	MPO	+	+		SAH	Renal Abdominal pains	-	MP 0.5 g	NR	-	Death
Sakura M et al. 2016^[21]^	64	F	Present	MPA	MPO	+	−		SAH	Renal	Renal Autopsy	Steroid pulse	-	-	Death
D Marnet et al. 2010^[22]^	63	F	4 years	GPA	PR3	+	+		SAH	Renal Skin Cystitis	Renal	MP	NR	-	Remission
M. C. VENNING et al. 1991^[23]^	36	M	6 months	GPA	-	−	−		SAH	Pulmonary Myalgia Arthritis	-	PNL	CTX	-	Remission
M. C. VENNING et al. 1991^[23]^	50	M	4 years	GPA	c-ANCA	+	−		SAH	Renal Pulmonary Skin	-	PNL	-	-	Remission
D N Cruz et al. 1997^[24]^	71	M	Present	GPA	p-ANCA	+	−		SAH	Renal Pulmonary	Renal	MP 1 g	CTX	-	Remission
S. Fomin, et al. 2006^[25]^	17	M	1 year	GPA	c-ANCA	+	−		SAH	Renal Pulmonary Skin Ventricular bleed	Skin	High dose	CTX	SAH	Death
R. Nardone et al. 2004^[26]^	78	F	NR	GPA	c-ANCA PR3	+		−	SAH	Pulmonary Skin Myocardial infarction	Autopsy	NR	NR	-	Death
J. Douglas Miles et al. 2011^[27]^	74	F	11.5 weeks	GPA	c-ANCA PR3	+		−	SAH Ventricle hemorrhage	PNS Renal Pulmonary Skin Arthritis Liver	Nasopharyngeal mass	MP	CTX	-	Death
Hiroyuki Takei et al. 2004^[28]^	34	M	NA	GPA	c-ANCA	+		+	SAH	PNS Pulmonary Skin	Renal	Steroid	CTX	-	Remission
Matilda X. W. LEE et al. 2017^[29]^	48	F	1 year	EGPA	MPO	+		+	SAH Ventricular hemorrhage	PNS Skin	Breast Nerve	MP1g	CTX	Intracranial hemorrhage	Death
J. M. Calvo-Romero et al. 2002^[30]^	47	F	6 years	EGPA	MPO	−		−	SAH	PNS Skin	Skin	PRED 1 mg/kg	CTX	-	Remission
Shigeyuki Sakamoto et al. 2005^[31]^	36	F	8 years	EGPA	-	+		+	SAH	PNS Gastroenteritis	-	PRED	-	-	Remission
Shogo Matsuda et al. 2018^[32]^	48	F	8 months	EGPA	MPO	+		−	SAH	PNS Skin Arthritis Cardiac ischemia	Skin	Betamethasone	AZA RTX	-	Remission
Cormac Southam et al. 2019^[33]^	56	M	1 year	EGPA	p-ANCA MPO	+		−	SAH Spinal SAH Ventricular hemorrhage	PNS Pulmonary	Nerve	MP	-	-	Poor/death
A.MALOON et al. 1985^[34]^	39	M	3 years	EGPA	NR	Initial-relapse +		−	SAH	Pulmonary Skin	Skin	PNL 80 mg	CTX	SAH	Death
Kyoko Shimizu et al. 2011^[35]^	60	F	9 years	EGPA	-	+		+	SAH	PNS Pulmonary Arthritis Phrenic nerve paralysis	-	PSL	CsA	-	Remission
L. Tyvaert et al. 2004^[36]^	47	F	1 month	EGPA	MPO	+		−	SAH Occipital hematoma	PNS Skin Myalgia Abdominal pains	Salivary gland	Steroid	NR	-	Remission
Luca Diamanti et al. 2014^[37]^	31	F	Long-term	EGPA	p-ANCA MPO	+		−	Spinal SAH	PNS Skin Arthritis	-	MP 1 mg/kg	RTX	-	Remission
Myeong Hoon Go et al. 2012^[38]^	39	M	9 months	EGPA	MPO	+		IVAD	SAH Ventricular hemorrhage	PNS Renal Pulmonary Skin Arthritis Pericardial effusion	Renal Skin	MP 1 mg/kg	CTX	-	Death
U.-M. Sheerin et al. 2008^[39]^	37	F	Present	EGPA	p-ANCA MPO	+		−	SAH	-	-	MP	-	-	Remission
V.G. Menditto et al. 2013^[40]^	64	F	6 years	EGPA	MPO	+		+	SAH	Skin	Skin	PRED 1 mg/kg	-	Aneurysm	Remission
Chang Y et al. 1993^[41]^	47	F	20 years	EGPA	NA	+		−	SAH	PNS Pulmonary Epigastric pain	-	PSL	CTX	-	Death
M Ito et al. 2014^[42]^	68	M	NR	EGPA	NR	+		CAD	SAH	PNS Arthritis	-	Steroid	-	-	Remission
Giuseppe Taormina et al. 2014^[43]^	58	M	7 years	EGPA	p-ANCA	+		−	SAH Cerebral infarction	Skin Coronary Artery Stenosis	Bone nasal	PRED 1 mg/kg	-	-	Remission
K Muraishi et al. 1988^[44]^	29	F	Present	EGPA	-	+		+	SAH Occipital hematoma	Renal	Aneurysm	Steroid	-	-	Remission
A. Lázaro Romero et al. 2021^[45]^	54	M	3 years	EGPA	-	+		−	SAH Spinal epidural hematoma	PNS Skin Asthma	-	-	-	-	Death
Mrackova J. et al. 2020^[46]^	52	F	A few months	EGPA	C-ANCA	+		−	SAH	Asthma Pulmonary Nasal polyposis	-	Corticosteroids	CTX	Intracranial hemorrhage	Remission
Lescuyer Sylvain et al. 2016^[47]^	43	M	3 years	EGPA	P-ANCA MPO	+		−	SAH Ventricular hemorrhage	Asthma Myalgia Arthritis Peroneal neuritis	-	MP 0.5 g	CTX	-	Remission
Tessa A. Harland et al. 2019^[48]^	48	F	4 months	AAV	P-ANCA MPO	−		+	SAH Spine SAH	Weakness Dysarthria Paresthesia	-	Steroids	RTX	-	Remission

Abbreviations: Dx, diagnosis; SI, systemic involvement; ANCA, anti-neutrophil cytoplasmic antibody; c-ANCA, cytoplasmic ANCA; p-ANCA, perinuclear ANCA; MPO, myeloperoxidase; PR3, proteinase3; EGPA, eosinophilic granulomatosis with polyangiitis; MPA, microscopic polyangiitis; GPA, granulomatosis with polyangiitis; IS, immunosuppressive; RTX, rituximab; CTX, cyclophospham; MMF, mycophenolate mofetil; AZA, azathioprine; CsA, ciclosporin; NR, not reported; PNL, prednisolone; PRED, prednisone; MP, methylprednisolone; CAD, cerebral artery dissection; IVAD, intracranial vertebral artery dissection

Patients with concomitant other CNS manifestations or cardiac abnormalities contributed substantially to the overall mortality. Ten patients had one or more cerebrovascular events, one with combined idiopathic late-onset cerebellar ataxia, two with cerebral infarction, six with ventricular hemorrhage, two with occipital hematoma, and spinal epidural hematoma in one patient. Cardiac abnormalities were observed in six patients with AAV and SAH, with four cases causing lethality.

SAH is often associated with a poor outcome, with a mortality rate of over 50%, irrespective of treatment [12]. In the case series, all patients with SAH had a mortality rate of 38.2%. Thirty-one patients were treated with glucocorticoids, and 18 patients also received immunosuppressive therapy. CTX was the most commonly used immunosuppressant. Three patients received rituximab (RTX) treatment and achieved remission. Patients with SAH benefited from combined therapy with corticosteroids and immunosuppressants. All cases of AAV with SAH had a mortality rate of 38.2% and benefited from combined therapy with corticosteroids and immunosuppressants. However, the data demonstrated that concomitant cerebrovascular events or cardiac involvement in patients with AAV and SAH could progressively deteriorate the prognosis with a mortality rate of 64.3%.

## Conclusion

Our study suggests that SAH is a rare severe manifestation and associated with active AAV, which should be considered in patients with AAV due to the high rate of fatality, even in patients with a negative CT scan. Early diagnosis and immunosuppressive therapy are crucial to achieving a favorable prognosis.

## Supplementary information

Below is the link to the electronic supplementary material.

Supplementary file1 (PDF 434 KB)
