# Supplementary material for: Relapsing subarachnoid hemorrhage as a clinical manifestation in microscopic polyangiitis: a case report and literature review
Source: Clin Rheumatol. 2022 Jun 11;41(10):3227–35. doi: 10.1007/s10067-022-06163-6 (PMC9485077; doi:10.1007/s10067-022-06163-6)
Supplement: Supplementary file 1 — Supplementary file1 (PDF 434 KB) [file 10067_2022_6163_MOESM1_ESM.pdf]

ELSEVIER

Certificate of Elsevier Language Editing Services

The following article was edited by Elsevier Language Editing Services:

"Relapsing subarachnoid hemorrhage as a clinical manifestation in microscopic polyangiitis□ A case report and literature review"

Authored by:

Jingjing Xie, Ertao Jia, Suli Wang, Ye Yu, Zhiling Li, Jianyong Zhang, Jia Li

Date: 01-Mar-2022
Serial number: LE-233658-B9DBE8C6091F
